# Owner-Observed Behavioral Characteristics in Off-the-Track Thoroughbreds (OTTTBs) in Equestrian Second Careers †

**DOI:** 10.3390/ani15142046

**Published:** 2025-07-11

**Authors:** Anne-Louise Knox, Kate Fenner, Rebeka R. Zsoldos, Bethany Wilson, Paul McGreevy

**Affiliations:** 1 UQ School of Agriculture and Food Sustainability, University of Queensland, Gatton, QLD 4343, Australia; kate.fenner@uq.edu.au (K.F.); rebeka.zsoldos@slu.se (R.R.Z.); 2 Department of Biosystems and Technology, Swedish University of Agricultural Sciences (SLU), 750 07 Alnarp, Sweden; 3 Sydney School of Veterinary Science, University of Sydney, Camperdown, NSW 2006, Australia; bethany.wilson@sydney.edu.au (B.W.); paul.mcgreevy@sydney.edu.au (P.M.)

**Keywords:** equine behavior, equestrian disciplines, racehorse welfare, racehorseretraining, racehorse rehoming, retired racehorse

## Abstract

The off-the-track Thoroughbred’s (OTTTB’s) suitability for equestrian retraining and rehoming should always be subject to behavioral considerations. Certain attributes may be advantageous to a horse’s racing performance but unfavorable to their prospects off the track. It is important to gain a non-biased understanding of how Thoroughbreds (TBs) in equestrian disciplines compare with other horses behaviorally, to minimize risks of poor welfare and safety outcomes. The current study used owner-reported information (*n* = 1633) from the Equine Behavior Assessment and Research Questionnaire (E-BARQ) global database to compare the behaviors of OTTTBs with those of other ridden horses. Boldness, compliance, rideability, trainability, and responsiveness to acceleration and deceleration signals were evaluated in the context of 27 E-BARQ items, as determined by exploratory factor analysis (EFA). In this study, OTTTBs demonstrated more boldness (*t* = 3.793; *p* < 0.001) and lower compliance and responsiveness to deceleration signals (*t* = 3.448; *p* < 0.001) than non-OTTTBs. Trainability, rideability, and responsiveness to acceleration signals did not differ significantly between OTTTBs and non-OTTTBs. These findings provide direction for future research into causal factors and improvement opportunities regarding the training and management of Thoroughbreds, on- and off-the-track.

## Simple Summary

Equestrians offer a pivotal avenue of rehoming for Thoroughbred horses that retire from the racing industry. However, behavioral attributes considered advantageous in racehorses may be inappropriate in equestrian mounts. Understanding the behavior of off-the-track Thoroughbreds (OTTTBs) is critical to the welfare and safety of horses and handlers. This study explored behavioral characteristics in OTTTBs compared with other ridden horses, using owner-observed information from the Equine Behavior Assessment and Research Questionnaire (E-BARQ) database. Behaviors associated with boldness, compliance, rideability, trainability, and responsiveness to acceleration and deceleration signals were analyzed. OTTTBs demonstrated more boldness, with lower compliance and responsiveness to deceleration signals, than other horse breeds in this study. These findings illustrate both the equestrian potential of OTTTBs and the behavioral challenges they can present. The study highlights the importance of further research into the relative influences of breed and life experiences on behavior in OTTTBs, to enable training and management practices to be evidence-based for enhanced equine welfare.

## 1. Introduction

Each year, over 4000 Thoroughbred (TB) horses leave the Australian racing industry [1]. Approximately one-third of these become breeding animals [1,2], while the remainder compete for a limited number of suitable new homes. With total horse ownership distributed among fewer than 400,000 Australians [3], and a reasonable equine life expectancy of more than 20 years, the ongoing supply of off-the-track Thoroughbreds (OTTTBs) far exceeds demand. Those suitable for equestrian purposes are likely to have more post-racing opportunities than others less suitable [4]. The most common equestrian use for OTTTBs is pleasure riding, with other popular activities including eventing, show-jumping, dressage, pony club, adult riding clubs, showing, and polo [5,6,7,8]. Optimizing demand for OTTTBs is contingent on promoting positive stories. However, given the risks of poor welfare and safety outcomes on one hand and unnecessary wastage of horses on the other, there is a need for targeted and objective research that can support responsible advocacy.

A horse’s suitability for a successful equestrian career depends on its behavioral attributes [9]. Behavior is one of the least-cited reasons for TBs to be withdrawn from a racing career [6,7,8], and retirement on behavioral grounds is largely reserved for horses that resist entering the starting gates at the racetrack [6]. Notably, being slow is not considered a behavioral attribute. This explains why a survey of TB trainers reporting on reasons for horses exiting the Australian racing industry in 2017–2018 determined behavior to be the reason in only 2.4% (*n* = 780) of cases [7]. In contrast, over one-third of OTTTB owners in the United States surveyed by Reed et al. (2020) reported unwelcome behaviors within the first year of the horse’s exit from racing, with around 25% (*n* = 1391) of these issues being riding-related [10]. This incongruity may reflect a divergence in how equine behavior is perceived and prioritized at the racing–equestrian interface. While performance potential is paramount in racing, research shows that equestrians, both professional and recreational, consider behavior to be the utmost priority when selecting a horse [11,12,13].

Racing is a high-adrenaline sport that harnesses the equine flight response. As a species, horses are physiologically well-adapted to flee from threats, with skeletal muscle composition, cardiovascular capacities, and neurological and metabolic responses superior to most other mammals [14]. These attributes are pronounced in the TB as a result of generations of selective breeding for speed [14,15,16,17,18]. While this endows the TB with the athleticism to excel as a performance horse both on- and off-the-track [19,20], it can manifest as behaviors undesirable to an equestrian rider [21,22]. The magnitude with which a horse responds to sensory stimuli impacts their ease of handling and riding, ability to adapt to novel circumstances, and commercial value in the equestrian market. Nevertheless, OTTTB enthusiasts are plentiful. The OTTTB owners surveyed by Reed et al. [10] were more than twice as likely as other horse owners to report behavioral issues, yet were overwhelmingly satisfied with their decision to acquire an OTTTB, with 97% saying they would do the same again and would recommend the decision to others.

Notwithstanding, whether patently dangerous or merely undesirable, unwelcome equine behaviors are associated with a decrease in value, an increased likelihood of relinquishment, and a decline in welfare [4,8,23,24,25,26,27,28]. OTTTBs have been overrepresented in surveys of unwanted horses in the United States [29,30], the United Kingdom [4], and Ireland [31]. This may simply reflect the primary surplus of OTTTBs, or it may also be symptomatic of a heightened vulnerability to unstable rehoming outcomes. For horses, unlike companion animals, it is quite normal to move through multiple homes during a lifetime as owners’ intentions and circumstances change [32,33,34,35]. In an Australian study of horses presented for sale at public livestock auctions, most TBs identified were not in racing condition, suggesting that they had not come directly from the track but rather from subsequent homes [36].

It is widely accepted that OTTTBs require considerable retraining and that even after retraining, some remain unsuitable for inexperienced riders [9,14,22]. In Safe Work Australia’s *Guide to Managing Risks When New and Inexperienced Persons Interact with Horses* [37], the TB is the only breed that receives specific cautionary mention. In dogs, unwelcome behaviors are a leading cause of relinquishment [38], and in comparison, equine rehoming is a more fraught undertaking. Horses need carers with a higher skill set than companion animals, and their behaviors are influenced by the abilities and limitations of their guardians [39]. The non-racing sector accounts for approximately 85% of the Australian domestic equine population [3], yet there is no skill, knowledge, or experience prerequisite for horse custodianship outside of racing [40]. In a 2019 study of why horses were relinquished to non-profit organizations in the UK, only 5.9% (*n* = 791) of respondents cited behavior as the reason. However, an additional 27% reported either the horse or the owner to be “unsuitable”, potentially signaling behavioral issues that may have gone unrecognized [4]. A 2012 survey of horses in rehoming facilities across the USA found that 56% (*n* = 280) required behavioral modification to be suitable for adoption [25]. It is possible that equine behavioral assessment requires expertise and objectivity beyond the capacity of the relinquishing owner [4,32].

Behavior varies between individuals as a result of both temperament and life experience. Equine temperament is predetermined by innate neurogenetic properties and is relatively stable over time, whereas behaviors are continually influenced by external variables such as training, management, and environment [18,21,23,41,42,43]. OTTTBs make a compelling subject for a behavioral study, sharing a pattern of life experiences that sets them apart from other horse breeds. Notwithstanding, little equine behavioral research has been conducted in this area. The OTTTB’s equestrian potential has been widely demonstrated in the field [20], and the rich history of OTTTBs in equestrian sport has generated a vast body of anecdotal knowledge of their behavioral characteristics. However, evidence-based information resources are relatively lacking. Most equine behavioral research defines TB subjects only by breed, thereby introducing an array of additional physiological, environmental, and educational variables with the potential to influence behavior. A 2016 longitudinal study of TBs transitioning off-the-track determined that the most accurate predictor of equestrian suitability was the behavior score given by a test rider a full year after horses had finished racing. The behavior score assigned by a jockey while horses were still racing had no significant statistical relationship with their subsequent equestrian suitability [9]. Indeed, respondents to the survey conducted by Reed and co-authors reported that, with minimal intervention, 95% of unwanted behaviors resolved within the first year off-the-track [10]. Given the compound challenges of novel educational expectations, physiological changes, and environmental upheaval imposed upon a newly retired TB, behavioral assessment during this phase of transition can be misleading [42,43].

In general, equine research has largely focused on behavior as an indicator of temperament; the latter being defined as the repeatable individual variation in behavioral responses across time and contexts [44]. Objective research methods for assessing equine temperament involve observation of behavioral responses to stimuli under controlled conditions, either alone or in combination with measurement of physiological parameters [16,43,45,46,47,48]. Through behavioral testing, researchers thus far have defined three consistent temperament dimensions in the horse: neuroticism (flight distance and latency to approach a novel object), extraversion (exploration of a passive human and ease of handling), and sociability (reaction to separation and isolation) [42,43]. Heritability has been estimated for some temperament traits. Neophobic behaviors, in particular, appear to be influenced by breed and sire line [43,49,50,51,52]. The emerging field of equine behavioral genetics has identified genes associated with aspects of temperament such as vigilance [53], neuroticism [54], and adaptation to stress [15]. However, the influence of neurological and behavioral gene selection on differentiation of the TB from other breeds remains undetermined [23].

Behavioral responses used in the assessment of equine temperament can be unstable over time and contexts, even under controlled experimental conditions [44]. Subjective research methods, such as surveys, can offer valuable insights into behavioral consistency over time and contexts, provided that assessment tools are validated [43]. The Equine Behavior Assessment and Research Questionnaire (E-BARQ), a citizen science project, is a validated equine behavior assessment instrument [55], with an established and ever-growing database of records. The E-BARQ was developed to provide an owner-reported data resource for large-scale studies of relationships among behavior, training, and management in horses. The E-BARQ is open to owners and caretakers of all types of horses and can be accessed online.

The current study uses data extracted from the E-BARQ database to compare OTTTBs with other horses across six behavioral traits considered desirable in an equestrian mount: boldness, compliance, responsiveness to acceleration signals, responsiveness to deceleration signals, rideability, and trainability. The purpose of this study is to identify behavioral commonalities that exist among OTTTBs and establish a baseline for further research into the influences of external and physiological factors. This information may help inform evidence-based approaches to the selection, education, and management of OTTTBs.

## 2. Materials and Methods

### 2.1. Ethics Committee Approval

This project was conducted under the approval of the University of Sydney Human Research Ethics Committee (approval number: 2020/326).

### 2.2. Questionnaire Design

The E-BARQ was developed in consultation with an international panel of equine professionals with expertise in a diversity of fields. It comprises a series of demographic questions describing the horse and their owner/handler, followed by two parallel sets of questions relating to behavioral observations made within the preceding six months. The questionnaire bifurcates according to whether or not the horse has been ridden/driven throughout this period [56]. All behavioral questions are presented in the form of a five-point Likert scale. Numerical values are assigned according to the frequency with which an action is observed (from “never” to “always”), the severity of the action (from “no signs” to “serious signs”), or the applicability of a statement (from “strongly agree” to “strongly disagree”). A sixth option of “not observed/applicable” is provided for every question. E-BARQ questions are deliberately structured in such a way that respondents report on their horses’ behaviors in a strictly observational, non-interpretational capacity. The E-BARQ was created using Qualtrics survey software [57] and can be viewed in Appendix 1 of *The Equine Behavior Assessment and Research Questionnaire (E-BARQ): How the domestic equine triad can advance ethical equitation* [56].

### 2.3. Data Collection

The E-BARQ is accessible online at https://e-barq.com (accessed on 10 February 2023). Respondents create an account on the website, through which they can save and return to view their results. Upon completion of the questionnaire, respondents receive a *Share-&-Compare* feedback graph displaying their horse’s results alongside those of the E-BARQ population [56]. Respondents can repeat the questionnaire at six-monthly intervals to track their horse’s progress over time.

For the purposes of the current study, recruitment was targeted at OTTTB enthusiasts to optimize the ratio of case to control group numbers. The project was promoted to special interest audiences via social media, and recruitment material was emailed to equestrian associations and racehorse rehoming organizations for distribution amongst their networks.

This study tests the null hypothesis that OTTTBs (*n* = 265) do not differ significantly from other horses (*n* = 1368) in certain behavioral traits, using exploratory factor analysis (EFA) and two-sample *t*-testing. A raw total of 3046 E-BARQ responses were subjected to EFA at the outset. The initial case group consisted of 490 purebred TBs that had raced, or been prepared or trialed for racing, and were now engaged in non-racing activities. All activities other than racing were classified as ‘equestrian’ for the purposes of this study. The initial control group consisted of 2556 equids of any breed other than purebred TB. Purebred Standardbreds were excluded, due to their primary use as a racehorse. Only horses that have been ridden or driven within the prior six-month period were included in the study. For those with more than one record, only the earliest chronological record was used.

### 2.4. Comparison of Case and Control Demographics

Age and gender of survey respondents, as well as sex of horse, were subjected to Fisher’s exact test for count data to examine associations between case and control groups. Age of horse was compared between case and control groups using the nonparametric Wilcoxon rank sum test with continuity correction to evaluate differences in medians, and statistical significance set at *p* ≤ 0.05.

### 2.5. Exploratory Factor Analysis

#### 2.5.1. Selection of Variables

EFA provides a means of analyzing factors that cannot be directly measured, such as behavioral traits, by identifying measurable variables that correlate with these factors. Development of the original E-BARQ involved rotated principal component analysis, followed by construction of a *Share-&-Compare* feedback graph. This graph displays 13 behavioral categories, each representing a series of questionnaire items [56]. From these questionnaire items, variables of relevance to the current study were selected for EFA based on their relevance to the six aspects of behavior being examined.

Relationships between individual variables were examined using a Kendall’s Tau correlation matrix. The Kaiser–Meyer–Olkin (KMO) measure of sampling adequacy was employed to evaluate the suitability of the data for factor analysis. The KMO function is from the Psych package of R statistical software version 2.3.6 [58]. Bartlett’s test of sphericity was applied to ensure that the correlation matrix was not random. The Bartlett function is from the R package EFAtools version 0.4.4 [59].

#### 2.5.2. Factor Extraction and Rotation

To determine the optimal number of factors for this study, Kaiser’s rule for eigenvalues was applied, and scree plot and parallel analysis were performed using the Psych package [58]. Factors underwent Oblimin and Varimax rotation to determine their corresponding variables. The E-BARQ items that loaded most strongly on Varimax rotation of four factors were retained. Internal consistency was measured using the Cronbach’s Alpha function from the Psych package [58]. For reporting purposes, Factors 1 to 4 were labeled according to the *Share-&-Compare* graph behavioral categories of the E-BARQ items that loaded strongly on each [56].

### 2.6. Hypothesis Testing

Factor scores were computed for hypothesis testing, using the Bartlett method from the Psych package [58]. To improve normality, square root + constant transformation was applied to Factor 1 and 2 scores, and logarithmic + constant transformation to Factor 3. Factor 4 scores underwent a (sqrt(c-x))* − 1 transformation to counteract negative skew.

For each of the four factors developed, a linear model was fitted to the factor scores using the lm function in R [60]. Explanatory variables for each model consisted of group (case vs. control), gender of respondent, age of respondent, sex of horse, and age of horse, with significance testing of terms conducted using F-tests. The age and sex of horse interaction was retained for Factor 4, having produced a comparatively low *p* value, while remaining interactions were dropped to generate simplified additive models for all factors.

The two-sample *t*-test and predictive linear modeling were employed to test the null hypothesis that there would be no significant differences between case and control factor scores. For each factor group, the *t* value quantifies the difference between the predicted case and control sample means. The *p* value signifies the probability of obtaining a *t* value equal to or greater than the observed value, assuming the null hypothesis to be true. For all model predictions, 95% confidence intervals were calculated to quantify the precision and uncertainty of the estimates.

## 3. Results

### 3.1. Sample Composition

The raw dataset of 3046 E-BARQ responses comprised 490 case OTTTBs and 2556 control horses. Within the control group, 48.7% were purebred horses representing 92 different breeds, with Quarter Horses (9.19%), Arabians (3.21%), and Dutch Warmbloods (3.09%) being the most prevalent. The remaining 51.4% were described as “crossbreed” or “other”.

Across the study sample, horses ranged in age from 1 to 36 years, with a mean age of 11.0 years for the case group and 11.7 years for the control (see Table 1). Geldings constituted 58.8% of the total and mares 39.0%, with the remainder being fillies, colts, stallions, and rigs (see Table 2). Similar sample composition was obtained by Anzulewicz et al. [61] in a 2021 E-BARQ analysis of the impacts of the sex of handlers and riders on equine behavior. In that study, the sample comprised 58% geldings and 38% mares.

Within the study sample, 89.8% of survey respondents were in the 18–64-year age range. Of respondents, 92.5% identified as female, aligning with the study conducted by Anzulewicz et al., in which that figure was 95% [61]. Additional demographic details are provided in Supplementary Tables S1 and S2 (see Supplementary Materials).

### 3.2. Comparison of Case and Control Demographics

The OTTTBs surveyed were slightly but significantly younger than the horses in the control group, with a significant difference between median ages found on nonparametric testing (*p* = 0.031). Geldings were overrepresented, and mares and stallions underrepresented, among the case OTTTBs (see Table 3).

Post hoc testing showed that respondents in the 55–64-year age range were underrepresented amongst OTTTB owners/handlers, whereas those under 18 years old were overrepresented (see Table 4). Female respondents were more common within the case group than the controls (*p* = 0.020) (see Table 5).

### 3.3. Exploratory Factor Analysis

#### 3.3.1. Selection of Variables

Variables of relevance to the current study comprised 45 E-BARQ items, corresponding to seven *Share-&-Compare* categories: *Working Compliance*, *Easy to Stop*, *Boldness*, *Novel Object Confidence*, *Forward Going*, *Rideability*, and *Trainability*. There was a high incidence of missing data for some selected items, thus variables with more than 500 missing values were removed from the study. This left 1633 horses (265 cases and 1368 controls) with complete records for the 35 remaining variables.

Kendall’s Tau indicated that the correlation between remaining variables was <0.90. The KMO measure of sampling adequacy was 0.83 overall, and each item exceeded at least 0.69, which was determined as acceptable. Bartlett’s test of sphericity produced a significant result at an alpha level of 0.05. Based on these tests, the remaining 35 variables of interest were considered suitable for factor analysis.

#### 3.3.2. Factor Extraction and Rotation

The Kaiser rule for eigenvalues suggested 10 factors, the scree plot 4, and parallel analysis 11. Factors underwent Oblimin and Varimax rotation to improve interpretability. Regardless of whether 4 or 11 factors were selected for Oblimin rotation, three questions loaded poorly (<0.40) on all components and were removed. The 27 items that loaded most strongly on Varimax rotation of 4 factors were retained (see Table 6).

With a coefficient >0.70 for all factors, Cronbach’s Alpha indicated no further items that could be dropped to improve reliability. Factors 1 to 4 were labeled according to the *Share-&-Compare* graph behavioral categories of the E-BARQ items that loaded most strongly on each. These were as follows: Factor 1: *Working Compliance* + *Easy to Stop*; Factor 2: *Boldness* + *Novel Object Confidence*; Factor 3: *Rideability* + *Forward Going*; Factor 4: *Trainability* (see Table 7).

The E-BARQ item *Aggression–verbal correction—under saddle* was an anomaly, loading poorly on Factor 1 despite belonging to the *Share-&-Compare* graph category *Working Compliance*. For the purposes of this study, it was reclassified under Factor 3: *Rideability* + *Forward Going*, where it loaded strongly.

It is important to note that Factors 1 to 3 represent negatively worded E-BARQ questions. Hence, a lower score is considered more favorable. The opposite is true for Factor 4, deriving from positively worded questionnaire items.

### 3.4. Hypothesis Testing

#### 3.4.1. Factor 1: Working Compliance + Easy to Stop

**Figure 1:**
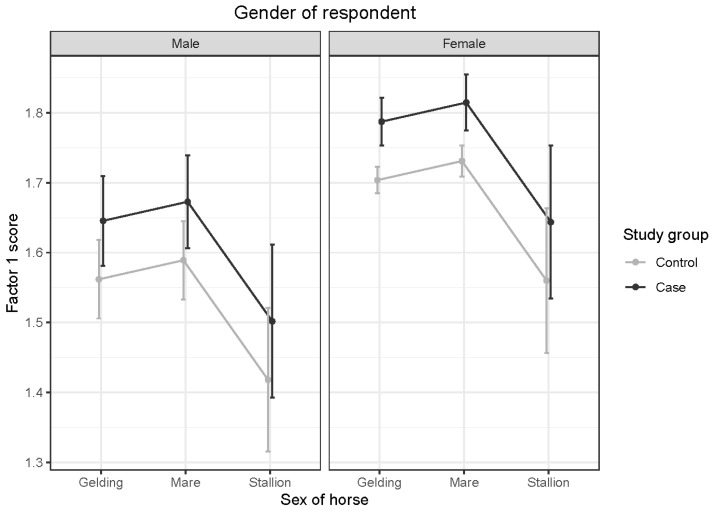
Predicted means with 95% confidence intervals form a linear model comparing case and control Factor 1 scores, shown by sex of horse, for (**left**) male and (**right**) female respondents. The higher scores obtained by case OTTTBs than control horses, and by female than male respondents, represent lower compliance and responsiveness to deceleration signals.

OTTTBs had significantly higher Factor 1 scores than control horses (*t* = 3.448; *p* < 0.001). As the E-BARQ items for Factor 1 were negatively worded, this finding indicates lower working compliance and responsiveness to deceleration signals in OTTTBs. Interactions between age and sex of horses and age and gender of respondents were not significant. The overall *p*-value for the sex of the horse was significant. However, pairwise comparisons of mare to gelding (*t* = −1.474; *p* = 0.304), stallion to gelding (*t* = 1.917; *p* = 0.134), and stallion to mare (*t* = 2.297; *p* = 0.057) failed to show significance. Female respondents reported significantly higher Factor 1 scores than male respondents (*t* = 3.786; *p* < 0.001). Given the negative wording of E-BARQ items, this indicates lower compliance and deceleration responsiveness in horses handled or ridden by female respondents (see Figure 1).

#### 3.4.2. Factor 2: Boldness + Novel Object Confidence

**Figure 2:**
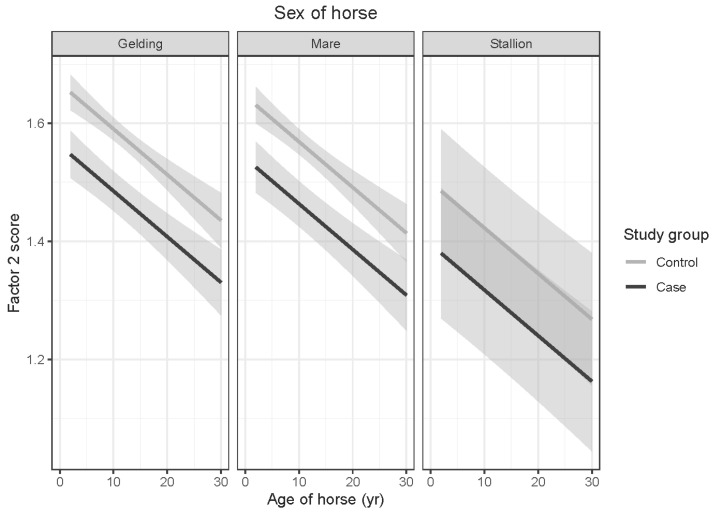
Predicted means with 95% confidence intervals form a linear model comparing case and control Factor 2 scores, shown by age of horse, for (**left**) geldings, (**middle**) mares, and (**right**) stallions. The lower scores obtained by case OTTTBs than control horses represent greater boldness and novel object confidence. The declining scores illustrate an age-related increase in boldness and novel object confidence for both case and control groups.

Factor 2 scores were significantly lower in OTTTBs than control horses (*t* = 3.793; *p* < 0.001). As the E-BARQ items for Factor 2 were negatively worded, this finding suggests a greater tendency towards boldness and novel object confidence in OTTTBs. Interactions between age and sex of horses and age and gender of respondents were not significant. Factor 2 scores declined significantly with age of horse (*t* = −3.713; *p* < 0.001). Given the negative wording of the E-BARQ items, this signifies an age-related increase in boldness and novel object confidence. While a difference between geldings and stallions was apparent, it did not reach statistical significance (*t* = 2.096; *p* = 0.091) (see Figure 2).

#### 3.4.3. Factor 3: Rideability + Forward Going

**Figure 3:**
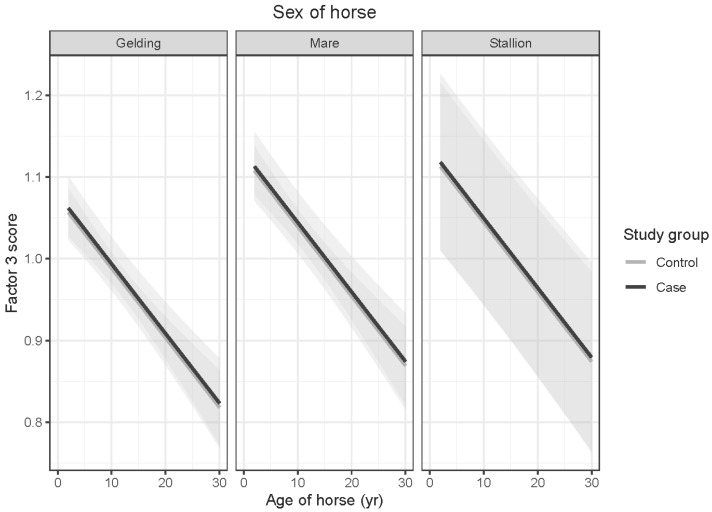
Predicted means with 95% confidence intervals form a linear model comparing case and control Factor 3 scores, shown by age of horse, for (**left**) geldings, (**middle**) mares, and (**right**) stallions. The declining scores illustrate an age-related increase in rideability and responsiveness to acceleration signals for both case and control groups.

**Figure 4:**
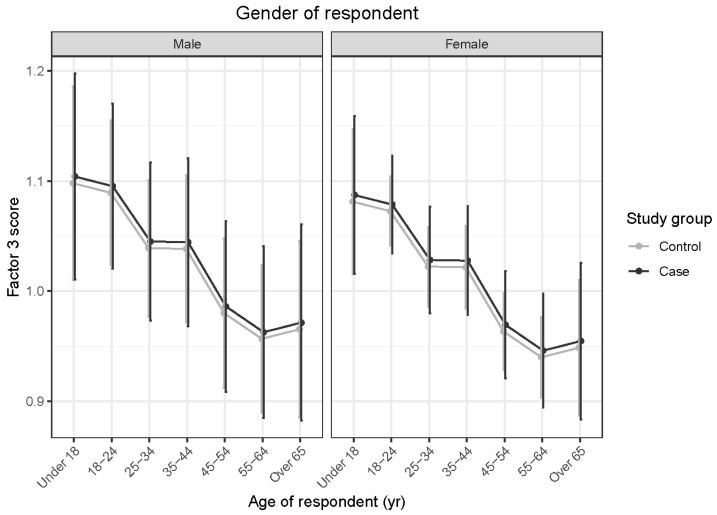
Predicted means with 95% confidence intervals form a linear model comparing case and control Factor 3 scores, shown by age of respondent, for (**left**) male and (**right**) female respondents. The lower scores obtained by respondents aged between 45 and 64 years, compared with those aged 18–24 years, indicate greater rideability and responsiveness to acceleration signals reported within the older age range.

OTTTBs did not demonstrate significantly different Factor 3 scores from the control horses (*t* = 0.190; *p* = 0.849). Interactions between age and sex of horses and age and gender of respondents were not significant. Factor 3 scores declined significantly with horse age (*t* = −4.417; *p* < 0.001). As the E-BARQ items for Factor 3 were negatively worded, this finding suggests an age-related increase in rideability and responsiveness to acceleration signals (see Figure 3). Geldings scored more favorably in these characteristics than mares, producing significantly lower Factor 3 scores (*t* = −2.486; *p* = 0.035). Respondents 45–54 years of age reported significantly lower Factor 3 scores than those aged 18–24 years (*t* = 3.357; *p* = 0.014), as did respondents in the 55–64-year age range (*t* = 3.883; *p* = 0.002) (see Figure 4).

#### 3.4.4. Factor 4: Trainability

**Figure 5:**
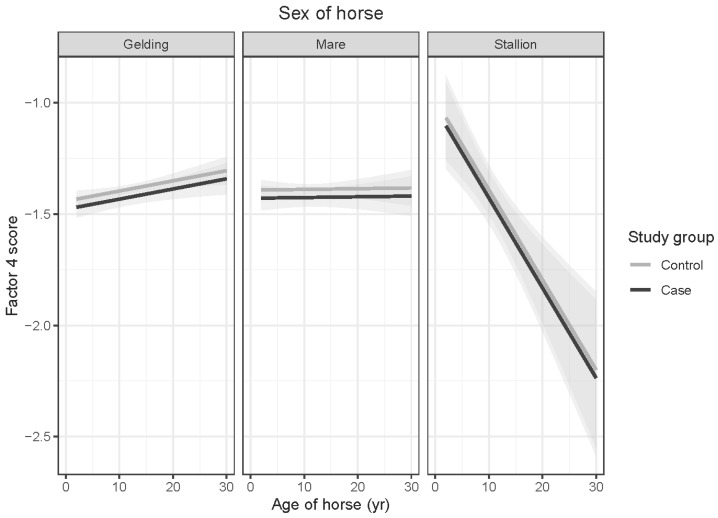
Predicted means with 95% confidence intervals form a linear model comparing case and control Factor 4 scores, shown by age of horse, for (**left**) geldings, (**middle**) mares, and (**right**) stallions. No significant correlations were identified between the age or sex of the horse and trainability.

**Figure 6:**
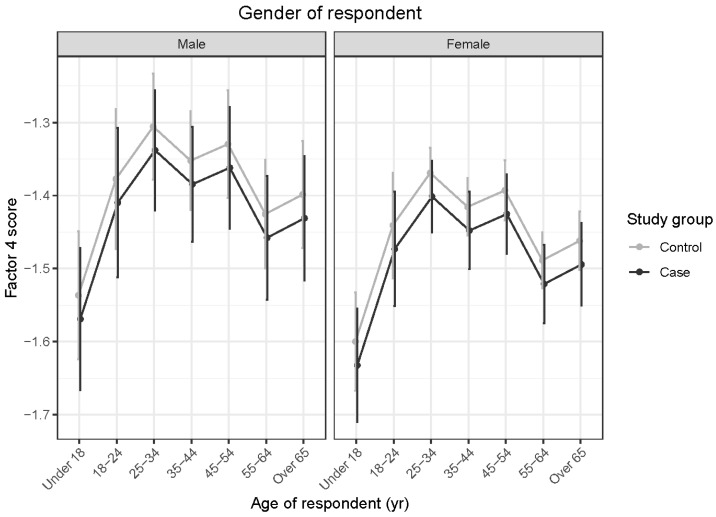
Predicted means with 95% confidence intervals form a linear model comparing case and control Factor 4 scores, shown by age of respondent, for (**left**) male and (**right**) female respondents. The higher scores obtained by respondents aged between 18 and 54 years, compared with those aged under 18 years, indicate greater trainability reported within the older age range. Likewise, the higher scores were obtained by 25–34-year-old than 55–64-year-old respondents.

Factor 4 scores for OTTTBs did not differ significantly from those for control horses (*t* = 1.112; *p* = 0.267). Interactions between age and sex of horses and age and gender of respondents were not significant. A difference in the effect of age on stallions and geldings was apparent but did not reach statistical significance (*t* = −1.732; *p* = 0.084) (see Figure 5). Respondents under the age of 18 reported significantly lower Factor 4 scores than those aged 18–24 years (*t* = −3.134; *p* = 0.029), 25–34 years (*t* = −4.441; *p* < 0.001), 35–44 years (*t* = −3.437; *p* = 0.011) and 45–54 years (*t* = −3.882; *p* = 0.002). Significantly lower Factor 4 scores were also reported by respondents in the 55–64-year age range than in 25–34-year-olds (*t* = 3.127; *p* = 0.030) (see Figure 6).

## 4. Discussion

### 4.1. Boldness

It is important to note that the E-BARQ items relating to *boldness* and *novel object confidence* are constructed in such a way that the level of boldness, in the context of this study, describes the absence of fear aversion rather than the presence of approach curiosity. The finding of significantly higher boldness in OTTTBs than control horses was unexpected. Objective research has shown TBs to have the most extreme behavioral and physiological responses of the three horse breeds subjected to a plastic bag test [62], and the second highest reactivity of 16 breeds subjected to a bridge test [50]. Furthermore, while TBs are widely incorporated into sport horse breeding programs to improve boldness [63] along with physical characteristics [49,63], combined testing has identified a positive correlation between fear reactivity and the proportion of TB in a sport horse’s pedigree [49]. Variation between experimental results and owner observations might be rationalized by the influence of horse–handler relationships on responses to novel objects, as this too has been demonstrated in behavioral and physiological tests [64]. However, other research suggests that a horse’s response to novel stimuli does not depend on their current handler’s identity, but rather on their general experiences with humans historically [65].

The boldness of OTTTBs compared with control horses in this study also challenges previous survey reports. While one study found no significant difference in boldness or nervousness between TBs, Arabians and Quarter Horses [66], others placed TBs among the highest-ranking of 13 breeds for nervousness and excitability [43] and of eight breeds for anxiousness and excitability [16]. This inconsistency of results may reflect different study designs, with a fundamental point of difference being that the current analysis strictly concerns OTTTBs in equestrian second careers. Notably, in one of the few contemporary studies that have examined OTTTBs in isolation from the wider breed population, OTTTBs were reported to have more “self-control” than horses bred for recreational riding [21]. The contrast in findings may also reflect different questionnaire approaches, with the E-BARQ focused on specific responses to stimuli rather than more general adjectival descriptors, and on owners’ observations of their horses’ behaviors rather than interpretations of their horses’ temperament.

However, there is further inconsistency between the findings of this and previous E-BARQ research. In a study of age-related behavioral changes, Burattini et al. (2020) analyzed 78 breeds individually and found TBs to be less bold than crossbred horses, heavy horses, and ponies [67]. Unlike the current study, this did not exclude TBs that were actively involved in racing and did include horses that had not been ridden within six months of survey participation. Given their perceived disposability compared with horses of other breeds, TBs lacking boldness may be less likely to enter or remain in ridden off-the-track careers. Moreover, the variables examined in each study were not equivalent. Research shows that equestrian horses with competition experience of any description exhibit significantly less general nervousness than those without [43], and, over the course of a racing career, OTTTBs might be expected to gain boldness similarly [21,68]. However, novel object tests indicate that habituation is object-specific, with horses being able to generalize only between objects that are very alike [69,70,71,72].

A common finding of both this study and that of Burattini et al. was the positive correlation between boldness and aging in both groups of horses. This aligns with other research evidence of an age-related decrease in reactivity [70] and an increase in “self-control” [21]. It is likely that the accumulation of exposure and habituation to stimuli over time is a key contributor to increasing boldness with age. However, it is also possible that advancing age may allow for longer duration of relationships with current handlers, a factor that positively influences novel object confidence in horses [65]. Paradoxically, the OTTTB group in the current study demonstrated significantly more boldness than the control group, despite being slightly but significantly younger. This may be an effect of TBs commonly starting under saddle as yearlings in preparation for two-year-old racing, younger than is typical in equestrianism. Research shows that an early introduction to ridden work is associated with greater boldness in most breeds [67]. It is likely that the accelerated and concentrated exposure of racehorses to stimuli and situations plays a role in the OTTTB’s comparative boldness.

While the findings of the current study suggest that OTTTBs may be less fear-averse than previously thought, further evaluation is crucial, given the safety implications of neophobic behavior. In Australia, horses are the leading cause of animal-related deaths [73], and equestrian activities have the fourth highest rate of sports-related hospitalization [74]. Ridden flight behaviors are the most significant risk factor in equestrian accidents and injuries [27,45,75,76,77]. Flight is the horse’s primary survival instinct. As an open-range prey species, equids coevolved with their predators by fleeing from danger. While modern domestic horses exist in quite different circumstances from their ancestors, they are frequently subjected to stimuli and situations that trigger the flight response [69,78]. This may range from generalized hyper-reactivity to full-scale bolting or, if escape is obstructed, anti-predator conflict behaviors, such as bucking and rearing [79]. Conversely, some horses adopt an initial passive coping strategy that is easily misinterpreted as calmness but can suddenly switch to active conflict behaviors if the horse is pushed beyond their arousal threshold [15,23,42]. Flight, like other fear-based responses, is more difficult to extinguish the more it is given expression [79,80]; a reality that is of particular relevance to OTTTBs. New South Wales became the first state in Australia to implement a workplace code of practice for the management of horse-related risk [81], following a fatal bolting incident at an agricultural college in 2009. The student who died was a relatively inexperienced rider; the horse, an OTTTB that had only recently retired from racing [82]. Appropriate preparation and pairing of rider and mount are imperative to mitigating the risk associated with equestrian activities in general [54,75,77], but particularly so when a horse has been bred and trained for heightened reactivity and flight responses [9]. There is a need for more comprehensive research in this area to support responsible retraining and rehoming of racehorses.

### 4.2. Compliance

Compliance, a term describing the consistency and correctness of a horse’s responses to trained cues from its rider or handler [61], was significantly lower among OTTTBs than control horses. This contrast was amplified by respondent demographics. The lower compliance found in OTTTBs triangulates with the concurrent findings of a significantly higher proportion of female riders and handlers among the OTTTB group than the control, and significantly lower compliance associated with female than male respondents. This third point aligns with previous E-BARQ research conducted by Anzulewicz et al. in 2021 [61], in which female riders and handlers reported significantly less compliance, such as head-tossing, bracing of the neck, and pulling on the reins or lead rope, than their male counterparts. Anzulewicz et al. [61] proposed that horses may be less inclined to trial such behaviors with male riders and handlers due to stronger rein or lead rope tension generated by greater physical strength. Another possible contributing factor may be a difference in competency distribution between genders. The horse-riding demographic is predominantly female at recreational and lower competitive levels, yet in elite equestrian sport, the ratio is typically reversed [3,83]. Hence, the greater compliance of horses ridden by males may be an effect of higher standards of equitation.

Non-compliant behaviors can be symptomatic of conflict, confusion, fear, habituation, or discomfort [84]. For example, raising the head to evade a rein cue, or to anticipate a canter transition, may indicate any number of issues from back pain to inappropriate choice of bit; from hyper-reactivity to inadequately trained acceleration and deceleration responses [84,85,86,87]. A priority when investigating any type of unwelcome equine behavior is to rule out underlying physical issues. In the context of the current findings, it is important to note that musculoskeletal injury is a leading cause for TBs being retired from racing; second only to poor performance [6,7,8,33]. While it is most often acute limb injury that ends a horse’s racing career [6,7], it is chronic, and frequently subclinical, back pathology that presents the greatest challenge to equestrian re-education [88,89,90,91]. A further consideration is that horses purpose-bred for equestrian sports may be exposed to more consistent physical therapies and management practices, such as saddle- and bit-fitting, dentistry, and bodywork. The E-BARQ provides scope for future examination of the efficacy of some of these factors.

Compliance in horses is also influenced by the methods and equipment used on them [61]. It must be acknowledged that the commercial nature of racing promotes a time-pressured and profit-driven approach to horse training. This can lead to education being foreshortened or substituted with harsher equipment, leaving deficits in the horse’s foundation training and cultivating non-compliant behaviors [79,92,93]. Training factors that lower compliance apply equally to equestrian activities as to racing [94,95]. It is possible that the lower monetary value placed on OTTTBs may subject them to lower standards of riding and training than other horses used for equestrian purposes. OTTTBs are relatively inexpensive to purchase [14]; a feature that may appeal particularly to lower-level equestrians. In the Australian commercial equestrian market, the TB is the only horse breed priced according to the sum of experience above all other factors [14]. In the sport horse domain, by contrast, experience is not a determinant of asking price and is instead negatively correlated with the likelihood of a successful sale [96]. Demand in the non-profit equine sector is greatest for horses that are suitable for beginner or intermediate riders, implying that advanced riders may be less inclined to acquire horses via rehoming channels [4,20,27,54]. Variables, such as duration and quality of off-the-track education, warrant future research.

### 4.3. Responsiveness to Deceleration and Acceleration Signals

Compared with the control horses in this study, OTTTBs were significantly less responsive to deceleration cues. This may reflect historic learning experiences involving pressure and release. While it might appear that speed is the only attribute required of a racehorse, trained deceleration responses are critical in racing, influencing the horse’s maneuverability and ability to conserve energy during a race [17,22]. However, there are nuanced differences between the horse’s learning experiences on and off the track that may contribute to the behaviors reported in this study. Most horse training methods are based on combined reinforcement concepts. In simplest terms, these involve application of a cue until the horse offers the correct response, then immediate removal of the cue (negative reinforcement), with or without provision of a reward (positive reinforcement). Timing is key to reinforcing the desired behavior; if a pressure cue is not removed when the correct response is offered, it acts instead as a deterrent [80,97,98,99]. For example, in most equestrian disciplines, a rider’s signal to slow down is applied via rein tension, and when the horse responds by decreasing its speed, the rider releases the rein tension. The rider may follow this with a positive reward, such as a scratch on the wither. Acceleration signals are delivered by the same principles of negative reinforcement, via pressure from the rider’s legs against the horse’s sides. However, leg cues are not an option when riding in the racing position, with very short stirrups. Instead, racehorses are commonly ridden with constant strong rein pressure, which, when released, signals the horse to accelerate [22]. When a rider maintains meaningless and relentless rein tension, it ceases to be negative reinforcement but rather acts as positive punishment. This desensitizes the horse to pressure signals and inhibits the deceleration response, training the horse to instead lean into the bit [80,97,98,99].

Likewise, the application of acceleration signals without meaningful escalation and timely release can lead to unresponsiveness [26,27,80,97,98,99]. The use of the whip in racing could be expected to have this effect. In Australia, there is no restriction on the frequency of whip use during the last 100 m of a race [100,101]. When the whip is applied to a horse that is already accelerating or fatigued, the whip acts as a source of positive punishment and inhibits learning of the acceleration response [79,93]. However, the current study found that OTTTBs were not significantly more or less responsive to acceleration signals than control horses. OTTTBs are not routinely exposed to the pressure of a rider’s legs on either side of their body until they embark on an equestrian second career [22]. This may preserve their responsiveness to acceleration signals delivered via a rider’s legs.

In E-BARQ research conducted by McKenzie et al. (2021), horses exposed to multiple handlers or riders were reported to have heightened deceleration responses and lessened acceleration responses, likely due to inconsistency between individuals’ cues [26]. McKenzie and co-authors proposed that horses may habituate more readily to leg and whip pressure than rein tension. However, this effect was not observed in the current study; perhaps due to the relative novelty of leg pressure cues for OTTTBs. In contradiction to this theory, Standardbreds were among the breeds least responsive to acceleration signals, with McKenzie et al. hypothesizing that this may be a result of their early training being in harness rather than under saddle [26]. A limitation of this study is that the E-BARQ items retained for analysis all related to rearing, one of the most extreme and least common manifestations of an inadequately trained acceleration response [76,95]. Other important items unrelated to rearing, such as not moving or moving backwards when given a forward cue, and responsiveness to acceleration signals delivered via whip or spurs, were excluded due to incomplete records.

### 4.4. Rideability

In the current study, no significant difference was found between OTTTBs and control horses in terms of rideability, a descriptor for the general quality of the riding experience. Overall, geldings scored more favorably in rideability and acceleration responsiveness than mares; a finding that may have advantaged OTTTBs, as geldings were overrepresented among this group. A limitation of the current study is that items describing the horse’s general responses to a rider’s seat, legs, and hands were excluded due to incomplete records, leaving only items relating to acute conflict behaviors involving defensiveness or aggressiveness, and bucking, pigrooting, or kicking out. Given the over-supply of OTTTBs and their relatively low commercial value in the equestrian market, it is possible that those with moderate or severe behavioral problems may be more readily culled from the population than horses of other breeds [26,27,84]. It is also possible that the campaign to recruit OTTTB enthusiasts for this project may have resulted in sampling bias towards those most inclined to seek evidence-based solutions to behavioral issues. The age-related improvement in both rideability and acceleration responsiveness identified in this study concurs with 2020 E-BARQ research conducted by Romness et al., which showed aging to be positively correlated with decreased bucking and rearing tendencies [102]. While this may be an effect of accumulated training, it may also reflect selective culling over time [21,27,67,102].

The current greater rideability and acceleration responsiveness reported by respondents 45–64 years of age compared with 18–24-year-olds may be a further by-product of the severity of behaviors analyzed. As they age, equestrians may assign increasing priority to behavior-related attributes in their selection of horses; likely due to a growing reluctance to risk injury [13]. A similar age-related aversion to real or perceived risk may be partially responsible for the underrepresentation of 55–64-year-old respondents and overrepresentation of 18–24-year-olds among OTTTB owners and handlers.

### 4.5. Trainability

Trainability refers to the ease and speed with which a horse learns the correct responses to human cues. No significant difference was evident between the trainability of OTTTBs and control horses surveyed. However, experimentally, TBs have demonstrated slower learning than other horse breeds when tested with a stepping-back task based on negative reinforcement [24]. While that finding does not align with the current findings on trainability, it does align with points discussed above in the context of deceleration. Ambiguity in the application of pressure cues during foundation training may predispose OTTTBs to becoming desensitized and disengaged with negative reinforcement-based learning [24]. A limitation of the current study is that questionnaire items relating to pressure cues were excluded due to incomplete records, leaving trainability to be evaluated in relation to voice cues only. While horses can learn to accommodate human voice cues, they are primarily reliant on non-vocal communication [103].

The biphasic OTTTB life trajectory makes the trainability of these horses an important focus. The typical retirement age for a TB racehorse of 5 years [6,7,104,105] is anecdotally regarded as ideal for retraining in new disciplines [19,20,106]. However, horses have remarkably long-term memory [23], and some evidence suggests that they have little capacity for reversal learning [78]. There may be an optimal window of time for foundation training, along with other key learning tasks, as various aspects of a horse’s behavior are shaped by experiences at specific developmental stages [67]. Ultimately, despite the diversity of activities that they are used for, all horses essentially only need to know four responses: stop, go, turn the forequarters, and turn the hindquarters. Horses with a clear and thorough foundation training in these basic responses are more easily educated and re-educated in different disciplines over the course of a lifetime [107,108]. In no equine demographic is this more critical than OTTTBs; a population defined by their collective career pivot.

### 4.6. Future Directions

The unexpected finding of greater boldness in OTTTBs than other horses in this study suggests that we should be cautious about generalizing our understanding based on research that examines the breed as a whole, rather than focusing specifically on TBs in equestrian activities. This underscores the need for more dedicated off-the-track behavioral research. With a better understanding of the impacts of a horse’s experiences on the track, these experiences might be optimized to benefit horses entering a second career.

### 4.7. Limitations

While behavioral testing under controlled conditions is a key facet of equine temperament research, citizen science can provide essential insight into how temperament, combined with life experience, manifests behaviorally in situ. With self-reported survey data, there is always potential for bias. For this reason, the E-BARQ is designed to minimize subjectivity by using only specific observations and recent recollections. Even so, it is possible that the targeted recruitment campaign to boost case numbers for this project may have resulted in sampling bias towards more dedicated OTTTB enthusiasts. Equine behaviors are complex in origin, arising from any combination of underlying factors associated with temperament and experience, along with physical health and soundness, and it is beyond the scope of this study to determine causality. However, the E-BARQ collects detailed information regarding training and management practices that could facilitate future analysis of the effects of these variables on behavior.

## 5. Conclusions

OTTTBs in this study using the E-BARQ database demonstrated more boldness, and lower compliance and responsiveness to deceleration signals, than other horse breeds. Trainability, rideability, and responsiveness to acceleration signals did not differ significantly between OTTTBs and other horses. There is a pressing need for more OTTTB-focused behavior research to help refine the selection, education, and management of horses for equestrian purposes, to better understand the impacts of their historic experiences, and to guide and strengthen the case for earlier integration of evidence-based training principles.

## Figures and Tables

**Table 1:** Age of horses in the case (OTTTB) and control groups.

Age of Horse (Years)	Case Group	Control Group
Mean	11.0	11.7
Median	10	11
Standard Deviation	4.6	5.7
Standard Error	0.2	0.1
Minimum	3	1
Maximum	31	36

**Table 2:** Sex of horses in the case (OTTTB) and control groups.

Sex of Horse	Case Group	Control Group	Study Sample	% of Total
Colt	0	2	2	0.1
Filly	0	13	13	0.4
Gelding	355	1430	1785	58.8
Mare	133	1053	1186	39.0
Rig	0	3	3	0.1
Stallion	0	49	49	1.6
Total			3038	

**Table 3:** Pairwise comparisons of the sex of horses in the case (OTTTB) and control groups. Statistically significant pairs (*p* ≤ 0.05) appear in bold.

Sex of Horse Comparison			*p*-Value
**Gelding**	**:**	**Mare**	**0.000**
**Gelding**	**:**	**Stallion**	**0.000**
**Mare**	**:**	**Stallion**	**0.037**
Filly	:	Gelding	0.317
Colt	:	Filly	1.000
Colt	:	Gelding	1.000
Colt	:	Mare	1.000
Colt	:	Rig	1.000
Colt	:	Stallion	1.000
Filly	:	Mare	1.000
Filly	:	Rig	1.000
Filly	:	Stallion	1.000
Gelding	:	Rig	1.000
Mare	:	Rig	1.000
Rig	:	Stallion	1.000

**Table 4:** Pairwise comparisons of the age of survey respondents in the case (OTTTB) and control groups. Statistically significant pairs (*p* ≤ 0.05) appear in bold.

Age of Respondent Comparison			*p*-Value	Age of Respondent Comparison			*p*-Value
**55–64**	**:**	**<18**	**0.000**	55–64	:	65–74	0.504
**18–24**	**:**	**55–64**	**0.000**	35–44	:	45–54	0.663
**35–44**	**:**	**55–64**	**0.003**	18–24	:	25–34	0.755
**25–34**	**:**	**55–64**	**0.003**	25–34	:	45–54	0.755
**45–54**	**:**	**55–64**	**0.030**	55–64	:	75+	0.755
**45–54**	**:**	**<18**	**0.038**	45–54	:	65–74	0.767
**65–74**	**:**	**<18**	**0.046**	75+	:	<18	0.863
25–34	:	<18	0.111	18–24	:	35–44	0.863
35–44	:	<18	0.183	18–24	:	75+	1.000
18–24	:	<18	0.267	25–34	:	35–44	1.000
18–24	:	65–74	0.267	25–34	:	75+	1.000
18–24	:	45–54	0.320	35–44	:	75+	1.000
35–44	:	65–74	0.418	45–54	:	75+	1.000
25–34	:	65–74	0.500	65–74	:	75+	1.000

**Table 5:** Pairwise comparisons of the gender of survey respondents in the case (OTTTB) and control groups. Statistically significant pairs (*p* ≤ 0.05) appear in bold.

Gender of Respondent Comparison			*p*-Value
Female	:	I’d rather not say	0.758
**Female**	**:**	**Male**	**0.020**
Female	:	Neither	0.776
I’d rather not say	:	Male	0.156
I’d rather not say	:	Neither	1.000
Male	:	Neither	0.328
Female	:	I’d rather not say	0.758

**Table 6:** Factor loadings on Varimax rotation. The strongest loadings, corresponding to the items that were retained on each factor, are shown in bold text. Shading intensity reflects the strength of loadings, with darker shading indicating stronger associations.

Item Question	Factor 1	Factor 3	Factor 2	Factor 4
Afraid—motorbikes	0.09	0.07	**0.68**	0.01
Afraid—cars	0.08	0.1	**0.68**	−0.01
Afraid—children	0.06	0.14	**0.47**	−0.02
Afraid—dogs	0.04	0.11	**0.46**	−0.07
Distracted—unfamiliar sights	0.25	0.08	**0.64**	−0.01
Distracted—unfamiliar sounds	0.22	0.11	**0.66**	−0.01
Responsive–voice cues—increase speed	−0.01	−0.09	−0.04	**0.79**
Responsive–voice cues—decrease speed	−0.22	−0.01	−0.06	**0.61**
Responsive–voice cues—change gait	−0.08	−0.01	−0.01	**0.79**
Aggressive–verbal correction—on ground	0.13	0.32	0.09	−0.11
Aggressive–verbal correction—under saddle	0.19	**0.42**	0.11	−0.07
Aggressive–canter cue—under saddle	0.2	**0.41**	0.05	−0.04
Raises head—rein/lead rope cues	**0.57**	0.2	0.08	−0.06
Tosses head—under saddle	**0.44**	0.25	0.05	−0.04
Pulls—rein/lead rope cues	**0.63**	0.19	0.05	−0.04
Braces neck—rein/lead rope cues	**0.55**	0.18	0.05	−0.03
Moves faster/raises head—canter	**0.55**	0.11	0.07	−0.03
Pulls forward—lead rope—walking	**0.44**	0.1	0.25	−0.07
Pulls forward—lead rope—trotting	**0.41**	0.12	0.25	−0.06
Pulls forward—lead rope—stop cue	**0.51**	0.12	0.19	−0.07
Pushes handler—led	0.35	0.18	0.17	−0.12
Pushes handler—food	0.33	0.24	0.07	−0.12
Backs up—forward cue	0.19	0.34	0.11	−0.02
Doesn’t move—forward cue	0.17	0.39	0.09	−0.14
Rears—forward cue	0.07	**0.57**	0.03	−0.02
Rears—under saddle	0.06	**0.61**	0.05	−0.03
Rears and flips—any time	0.04	**0.44**	0.02	0
Fails to slow—rein/lead rope cue	**0.68**	0.1	0.07	−0.01
Fails to stop—rein/lead rope cue	**0.68**	0.14	0.06	−0.03
Bucks—canter cue	0.22	**0.53**	0.1	0.06
Bucks—under saddle	0.18	**0.59**	0.16	0.08
Bucks—unseats rider	0.14	**0.57**	0.13	0.05

**Table 7:** E-BARQ *Share-&-Compare* graph behavioral categories and questionnaire items corresponding to each factor.

Factor 1: Working Compliance + Easy to Stop
*Does [Field–horsename]:* Raise head to avoid rein or lead rope cues; Toss head when being ridden/driven; Pull on reins or lead rope when signals are applied; Brace neck when rein or lead rope signals are applied; Move faster or raise head when anticipating the transition to canter; Fail to slow when signaled by a rein or lead rope cue; Fail to stop when signaled by rein or lead rope cue. *When on a lead rope in a familiar or typical situation, does [Field–horsename] pull:* Forward when walking; Forward when trotting; Forward when signaled to stop.
**Factor 2: Boldness + Novel Object Confidence**
*During the past 6 months, has [Field–horsename] been afraid of the following?* Motorbikes/quad bikes/ATVs/tractors; Cars/trucks/trailers; Children; Dogs. *Does [Field–horsename]:* Get distracted easily by unfamiliar sights; Get distracted easily by unfamiliar sounds.
**Factor 3: Rideability + Forward Going**
*Some horses display defensive or aggressive behavior in certain* *situations…Check a box on the five-point scale below to indicate* *[Field–horsename]’s recent tendency…to show these behaviors:* When verbally corrected when ridden/driven; When signaled to canter under saddle/in harness. *Does [Field–horsename]:* Rear when signaled to go forward; Rear up under saddle; Rear up and flip over at any time; Buck, pigroot, or kick out when signaled to canter; Buck at other times (when ridden); Unseat rider when bucking, pigrooting, or kicking out.
**Factor 4: Trainability**
*When ridden or driven, [Field–horsename] is responsive to:* Voice cues to increase speed; Voice cues to decrease speed; Voice cues to change gait.

## Data Availability

The original contributions presented in this study are included in the article/Supplementary Materials. Further inquiries can be directed to the corresponding author(s).

## Supplementary Materials

The following supporting information can be downloaded at: https://www.mdpi.com/article/10.3390/ani15142046/s1, Table S1. Age of survey respondents in case (OTTTB) and control groups; Table S2. Gender of survey respondents in case (OTTTB) and control groups.

## Abbreviations

The following abbreviations are used in this manuscript:

TB	Thoroughbred
OTT	Off-the-Track
OTTTB	Off-the-Track Thoroughbred
E-BARQ	Equine Behavior Assessment and Research Questionnaire
EFA	Exploratory Factor Analysis
KMO	Kaiser–Meyer–Olkin
