# Supplementary material for: Owner-Observed Behavioral Characteristics in Off-the-Track Thoroughbreds (OTTTBs) in Equestrian Second Careers
Source: Animals (Basel). 2025 Jul 11;15(14):2046. doi: 10.3390/ani15142046 (PMC12291702; doi:10.3390/ani15142046)
Supplement: Supplementary file 1 [file animals-15-02046-s001.zip › Supplementary Table S1.pdf]

Supplementary Table S1. Age of survey respondents in case (OTTTB) and control groups.

Age of Respondent (Years)	Case Group	Control Group	Study Sample	% of Total
<18	30	86	116	5.0
18–24	99	429	528	22.7
25–34	70	346	416	17.9
35–44	64	304	368	15.8
45–54	61	348	409	17.6
55–64	31	338	369	15.8
65–74	14	102	116	5.0
>75	1	7	8	0.3
Total			2330	
