# Supplementary material for: Owner-Observed Behavioral Characteristics in Off-the-Track Thoroughbreds (OTTTBs) in Equestrian Second Careers
Source: Animals (Basel). 2025 Jul 11;15(14):2046. doi: 10.3390/ani15142046 (PMC12291702; doi:10.3390/ani15142046)
Supplement: Supplementary file 1 [file animals-15-02046-s001.zip › Supplementary Table S2.pdf]

Supplementary Table S2. Gender of survey respondents in case (OTTTB) and control groups.

Gender of Respondent	Case Group	Control Group	Study Sample	% of Total
Female	353	1805	2158	92.5
Male	11	137	148	6.3
I'd rather not say	4	13	17	0.7
Neither	2	7	9	0.4
Total			2332	
